# Investigation of trends in gut microbiome associated with colorectal cancer using machine learning

**DOI:** 10.3389/fonc.2023.1077922

**Published:** 2023-03-01

**Authors:** Chaoran Yu, Zhiyuan Zhou, Bin Liu, Danhua Yao, Yuhua Huang, Pengfei Wang, Yousheng Li

**Affiliations:** Department of General Surgery, Shanghai Ninth People’s Hospital, Shanghai Jiao Tong University School of Medicine, Shanghai, China

**Keywords:** colorectal cancer, microbiome, bibliometric, Latent Dirichlet Allocation, Web of Science

## Abstract

**Background:**

The rapid growth of publications on the gut microbiome and colorectal cancer (CRC) makes it feasible for text mining and bibliometric analysis.

**Methods:**

Publications were retrieved from the Web of Science. Bioinformatics analysis was performed, and a machine learning-based Latent Dirichlet Allocation (LDA) model was used to identify the subfield research topics.

**Results:**

A total of 5,696 publications related to the gut microbiome and CRC were retrieved from the Web of Science Core Collection from 2000 to 2022. China and the USA were the most productive countries. The top 25 references, institutions, and authors with the strongest citation bursts were identified. Abstracts from all 5,696 publications were extracted for a text mining analysis that identified the top 50 topics in this field with increasing interest. The colitis animal model, expression of cytokines, microbiome sequencing and 16s, microbiome composition and dysbiosis, and cell growth inhibition were increasingly noticed during the last two years. The 50 most intensively investigated topics were identified and further categorized into four clusters, including “microbiome sequencing and tumor,” “microbiome compositions, interactions, and treatment,” “microbiome molecular features and mechanisms,” and “microbiome and metabolism.”

**Conclusion:**

This bibliometric analysis explores the historical research tendencies in the gut microbiome and CRC and identifies specific topics of increasing interest. The developmental trajectory, along with the noticeable research topics characterized by this analysis, will contribute to the future direction of research in CRC and its clinical translation.

## Introduction

Much of what makes colorectal cancer (CRC) studies cutting-edge has already been contributed by classic oncological approaches. But certain elements were lacking until the gut microbiome supplied them. What the gut microbiome contributes to carcinogenesis and tumor progression may be familiar to other research topics, but what the gut microbiome achieves in microbes’ contribution to this field is even more exceptional. A large population of microorganisms accommodated by the gut constantly interacts with intestinal epithelial cells and influences the metabolome and immunity throughout the entire gastrointestinal tract (1–5). Unbiased microbiome profiling and relevant models have revealed mechanistic insights into the microbial features associated with CRC (1). Remarkable progress has been achieved in studies relating to gut microbiota and CRC, highlighting the distinguished value of diagnosis and therapeutic prediction of the gut microbiome (6, 7). Bacterial strains such as *Fusobacterium nucleatum*, *Escherichia coli*, and *Bacteroides fragilis* are known for their tumor activities (8–10). However, there are many uncertainties concerning the association between gut microbiome and CRC, such as the huge number of bacteria (approximately 100 million) and distinct microbiota signatures, bacterial interactions, as well as geographical and race differences (11). Up to now, the gut microbiome remains far from fully deciphered.

Artificial intelligence (AI) has been one of the mainstays of cancer research and opens a plethora of technical applications (12). Driven by algorithms such as convolutional neural networks, a large quantity of data was used for training and pattern identification. It is commonly used to extract digital information from medical images for accurate medical diagnosis, such as MRI and PET/CT (13, 14). Meanwhile, mucosal visualization and polyp detection in gastrointestinal endoscopy and hematoxylin–eosin-stained images in pathological diagnosis can also be facilitated by AI (15, 16).

What occurred was so promising that, until recently, researchers began to discuss AI and the microbiome for colorectal cancer (17, 18). It is possible, however, to understand the value of the gut microbiome in CRC *via* an AI-dependent approach, and it is well worth while to do so. Based on megagenomic data and the antibiotic resistance genomic database, a DeepARG model was developed for accurate antimicrobial resistance annotation (19). Another example was a machine-learning-based decision tree model for prediction of cancer therapeutic responsiveness by gut microbiota composition and functional repertoire (20). Increasing volumes of metagenomics data and communications propel AI research intensity as well as platform-based data management and reusability (21).

Of note, AI is currently enriched in data-sensitive scenarios, but rarely covers text-sensitive scenarios of the gut microbiome. The rapid growth of publications on this research topic makes it feasible for text mining and bibliometric analysis. However, most analysis of research trends is mainly performed by literature reviews or meta statistics, with most word information untouched. Since a huge amount of wordy data over the decades could be a formidable task for manual processing, AI techniques are therefore translated for unbiased interpretation. Latent Dirichlet Allocation (LDA) is one of the most powerful machine learning-based approaches to text mining (22–24). It aids in the topics and relationship findings among publications and data. Therefore, this study was performed to characterize the research topics in the field of gut microbiome associated with CRC over the past twenty years. Topic modeling by LDA enables us to pinpoint each research topic and provide a more in-depth interpretation. Thus, the results of this study provide the developmental trajectory of this field, understandable sub-fields, or topic connections, as well as how each research topic branches out and multidisciplinary integration.

## Materials and methods

Publications in the field of gut microbiome with CRC were screened and retrieved *via* the Web of Science Core Collection (https://clarivate.com/webofsciencegroup/solutions/web-of-science-core-collection/) with search terms covering both CRC and microbiome from 2000 to 30 June 2022. All included publications were then processed for bibliometric extraction and citation analysis. Analysis software included R (4.1.1 version), CiteSpace (5.8 R3), and Gephi (0.9.5 version) (25–29). To identify specific research topics derived from all publications with insight, LDA, a machine learning-based algorithm, was used for text mining (30, 31).

## Results

**Figure 1:**
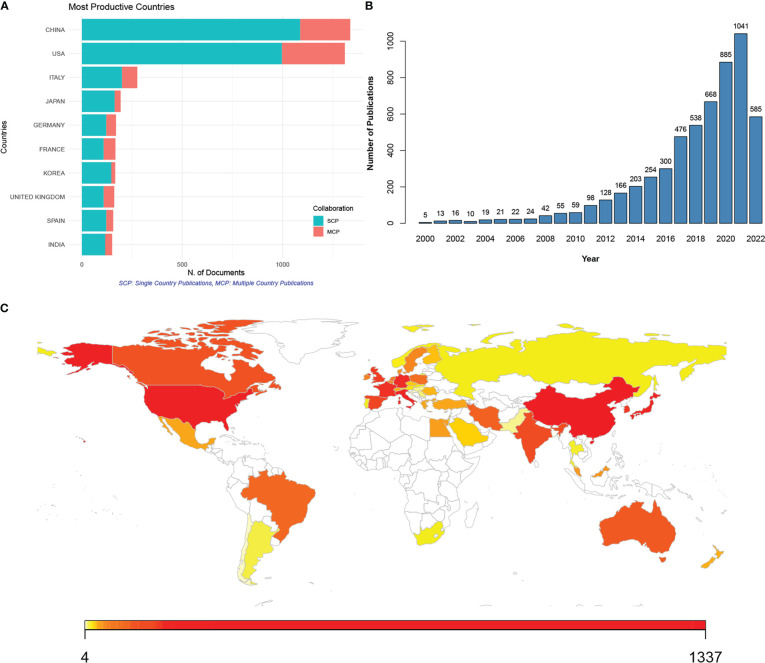
Publications of studies on the gut microbiome associated with colorectal cancer (CRC) from 2000 to 2022. **(A)** Top 10 countries with most publications of gut microbiome in CRC; SCP (green), single country publication; MCP (red), multiple country publication; **(B)** annual publications from 2000 to 2022; **(C)** contributing countries visualized by map; the number of publications was marked by color, with red to yellow indicating high to low publications.

A total of 5,696 publications related to the gut microbiome and CRC were retrieved from the Web of Science Core Collection from 2000 to 2022 (**Figures 1A–C**). The most productive countries included China, the USA, Italy, Japan, Germany, France, Korea, the UK, Spain, and India (**Figure 1A**). Although China published the most publications, the most multi-country publications were contributed by the USA. Meanwhile, a steady increase in annual publications was noticed (**Figure 1B**). Particularly since 2018, the increment of publications in each year has reached over 100.

**Figure 2:**
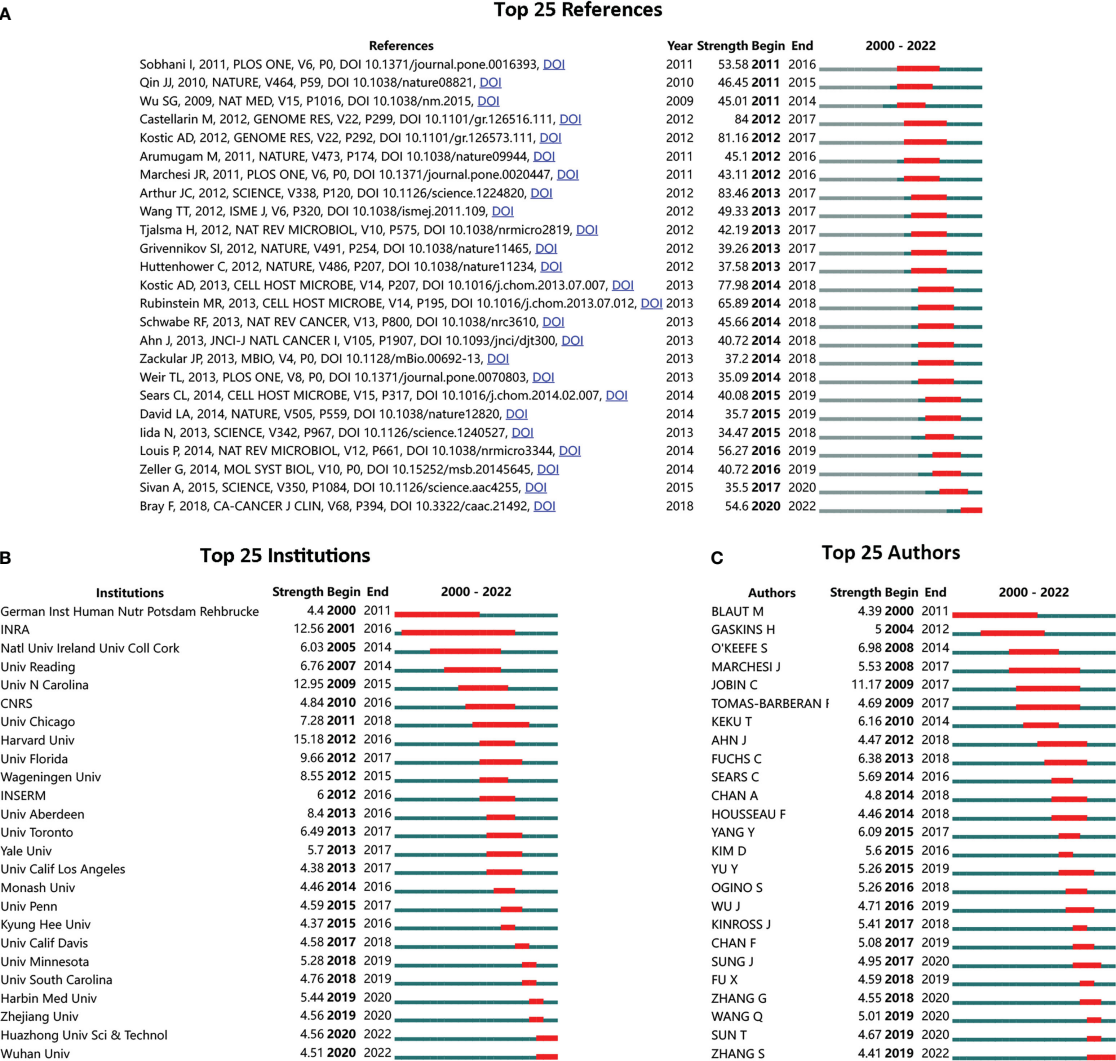
Top-listed references, institutions, and authors with the strongest citation bursts (SCB). **(A)** Top 25 SCB references; **(B)** top 25 SCB institutions; **(C)** top 25 SCB authors; red bar to green bar indicates high frequent occurrence of citation period compared to common frequent citation occurrence.

To further demonstrate the most influential references, institutions, and authors, bibliometric analysis was performed on all 5,696 publications. The top 25 references with the strongest citation bursts (SCB) were identified from 2000 to 2022. The reference with the highest strength was published in 2012 by Arthur JC in SCIENCE, “Intestinal inflammation targets cancer-inducing activity of the microbiota” (**Figure 2A**). The top 25 institutions and authors with SCB were also demonstrated. Harvard University was the top institution with the highest strength (**Figure 2B**). Meanwhile, the French National Institute for Agricultural Research (INRA) displayed the longest and strongest citation burst period, ranging from 2001 to 2016. Jobin C was the top author with the highest strength (**Figure 2C**).

**Figure 3:**
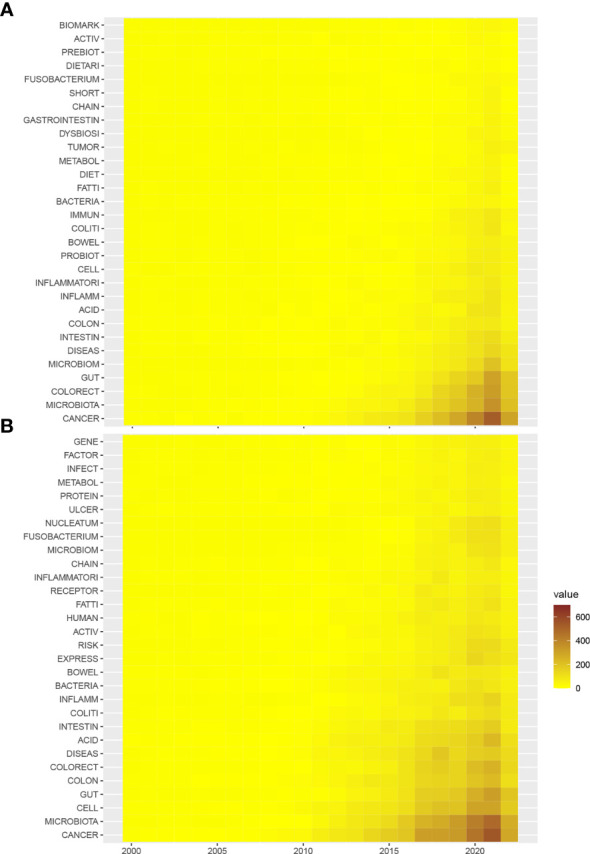
Heatmap of the annual occurrence of top keywords derived from the gut microbiome in the CRC. **(A)** Annual occurrence of author keywords from 2000 to 2022; **(B)** annual occurrence of keywords plus from 2000 to 2022.

Next, to further characterize the changes in keywords, the annual counts of keywords, both author keywords and keywords plus, were calculated from 2000 to 2022 (**Figures 3A, B**). In the author keywords such as fatty acid, inflammation, probiotics, colitis, diet, immune, metabolism, tumor, dysbiosis, and fusobacterium, biomarkers were among the top lists across the past twenty years. In keywords plus, not only similar words in author keywords were identified, but also keywords such as receptors, nucleatum, protein, and gene were added. In fact, the top key words can be categorized into four terms: microbiota composition (such as fusobacterium, nucleatum, and dysbiosis), microbiota and metabolism (such as metabolism, fatty acids, and protein), microbiota and treatment (such as prebiotics and probiotics, diet), and microbiota and disease course (such as tumor, biomarkers, activity, colitis, immune, infection, and risk).

**Figure 4:**
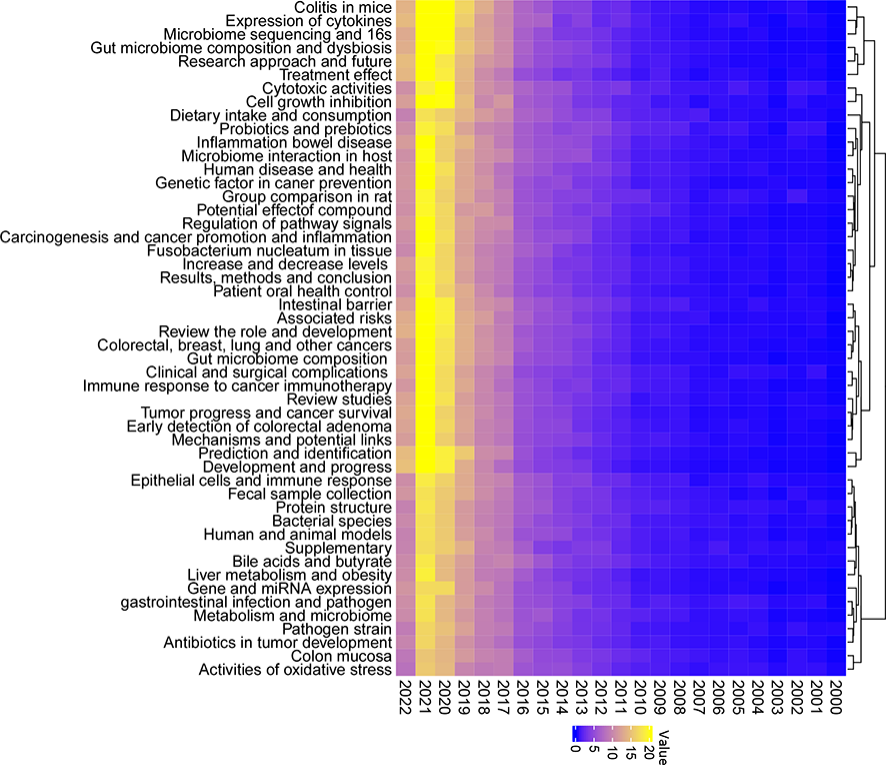
Fifty topics identified by the Latent Dirichlet Allocation (LDA) algorithm in publications of the gut microbiome in the CRC from 2000 to 2022. Blue, low occurrence value; yellow, high occurrence value.

To fully characterize the most investigated fields and to make research categorizations more precise within the gut microbiome and CRC, a LDA algorithm was employed. Abstracts from all 5,696 publications were extracted for a text-mining analysis. The results identified the top 50 topics in this field with increasing interest (**Figure 4**). In fact, topics such as colitis in mice, expression of cytokines, microbiome sequencing and 16s, gut microbiome composition and dysbiosis, and cell growth inhibition were dramatically increased during the last two years. Reasonably presumed, increasing studies in those subfields have achieved remarkable progress relating to CRC and gut microbiota.

**Figure 5:**
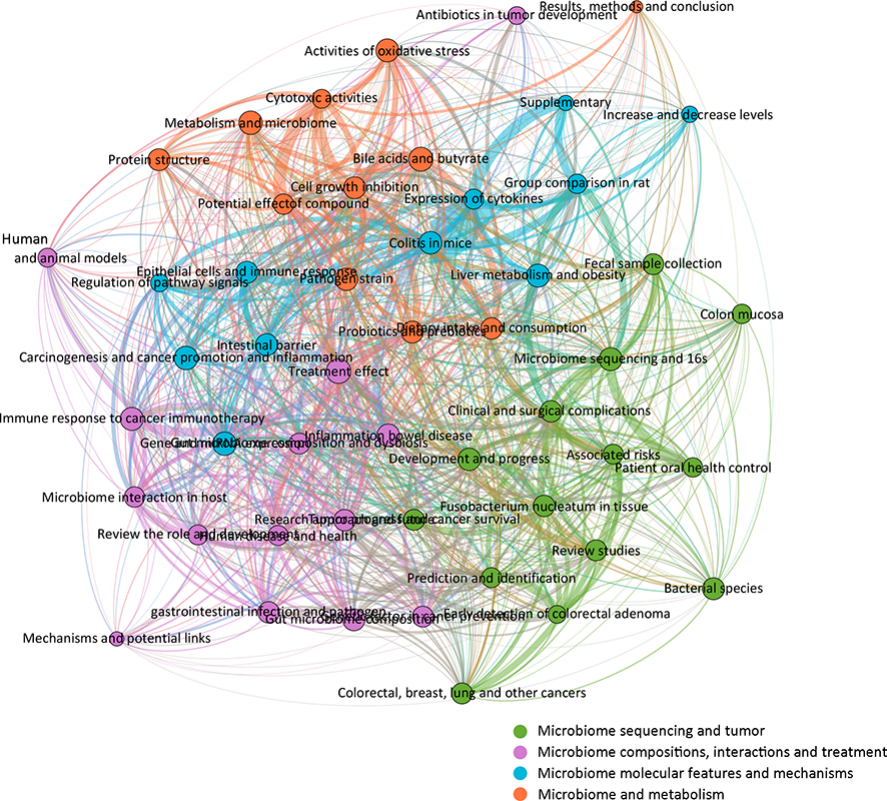
Network correlational and cluster analysis of the 50 topics. Four main clusters were identified and colored, respectively. Green, microbiome sequencing and tumor; violet, microbiome compositions, interactions, and treatments; blue, microbiome molecular features and mechanisms; orange, microbiome and metabolism.

To further complement the categorization of keywords aforementioned, the 50 topics were also categorized into several groups for analysis and developmental management. A total of four clusters were determined and colored, including “microbiome sequencing and tumor,” “microbiome compositions, interactions, and treatment,” “microbiome molecular features and mechanisms,” and “microbiome and metabolism” (**Figure 5**). The cluster “microbiome sequencing and tumor” was established by topics including microbiome sequencing and 16s, bacterial species, fecal sample collection, prediction, and identification of various tumors. The cluster “microbiome compositions, interactions, and treatment” was established by topics including human and animal models, treatment effects, immune responses to cancer immunotherapy, and others. The cluster “microbiome molecular features and mechanisms” was established by topics including expression of cytokines, carcinogenesis and cancer promotion and inflammation, regulation of pathway signals, and others. The cluster “microbiome and metabolism” was established by topics including dietary intake and consumption, bile acids and butyrate, activities of oxidative stress, and others. Based on those categorizations, several study areas for future studies have also been listed (**Table 1**), including four major areas: investigation models, microbiome sequence techniques, clinical trials, and microbiota metabolism.

**Table 1:** Gut microbiome research for future guidance.

Study area	Research topics
Human or animal models	1. Optimize the humanized microbiota-associated animal models 2. Establish animal model study on response to immunotherapy 3. Improving imaging techniques, such as animal endoscopy or MRI or tumor-specific markers
Microbiome sequencing	1. Machine learning application in next generation sequencing for gut microbiome 2. Microbiota dysbiosis and causes 3. Sample collections for sequencing
Clinical trials with gut microbiota	1. Specify the contribution of fecal microbiota transplantation (FMT) 2. Specify the association between FMT and immunotherapy 3. Application of microbial ecosystem therapeutics and its mechanisms 4. Association between probiotics and microbiota
Microbiota metabolism	1. Gut microbiota-derived short-chain fatty acids 2. Diet researches and tumorigenesis 3. Chemical-microbiota interaction for precancerous study

**Figure 6:**
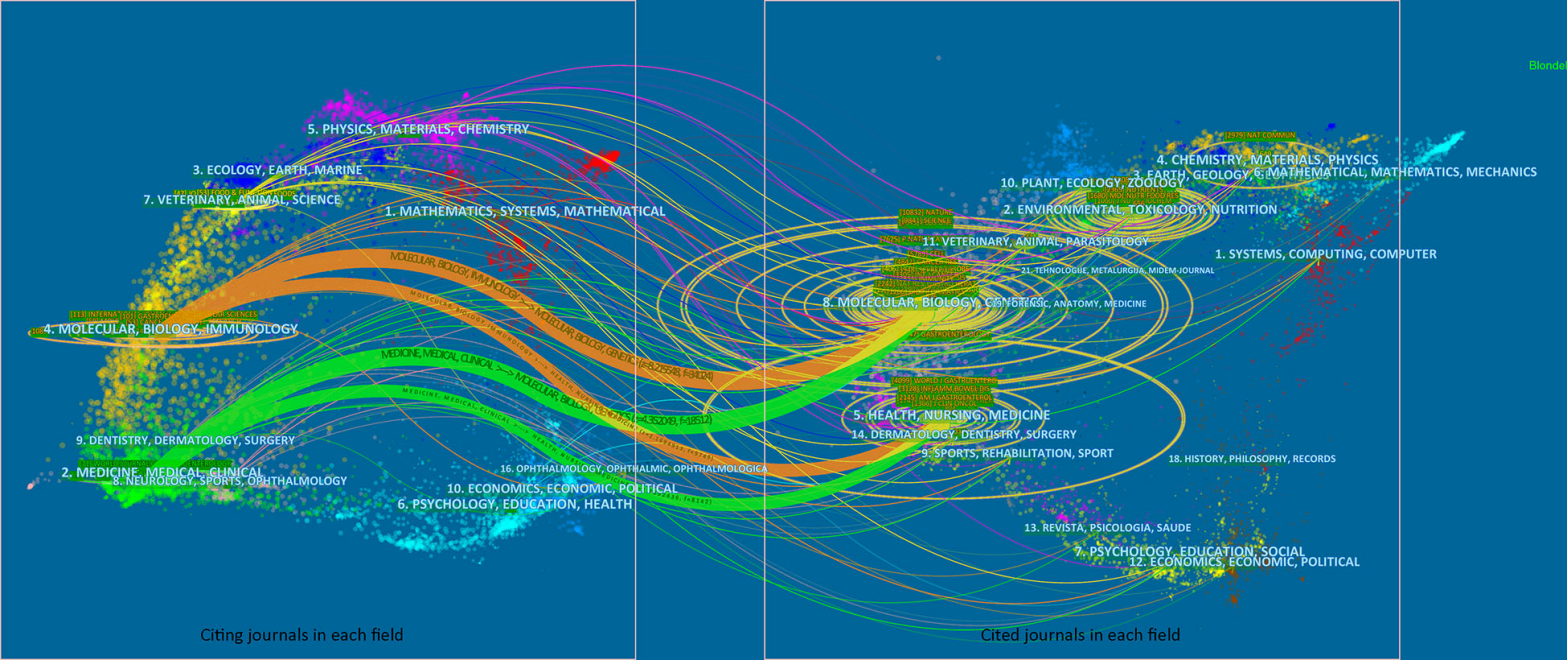
Dual-map citation portfolio analysis with thematic overlays. The publications and cited results were visualized. Wider edges a indicated higher value in occurrence. The left part of the plot indicated citing journals, and the right part of the plot indicated cited journals.

To visualize the citation pattern, a dual-map thematic overlay portfolio analysis was performed. The discipline distribution of publications associated with the gut microbiome and CRC was represented (**Figure 6**).

## Discussion

Up to 2021, the publication of gut microbiome in CRC has significantly increased by 200 times compared to 2000. A faster growth pace was found from 2015 to 2021, with significant increments in 2017 and 2020. The 50 predominant research topics identified in this study were clustered into four, including “microbiome sequencing and tumors,” “microbiome compositions, interactions, and treatment,” “microbiome molecular features and mechanisms,” and “microbiome and metabolism.”

Regarding microbiome sequencing and tumors, research is intensely focused on *F. nucleatum* in tissue, disease development and progress, microbiome sequencing and 16S, bacterial species, CRC, and colorectal adenoma. *F. nucleatum* had been reported to be closely associated with tumor subtypes as it induced diverse immune responses with respect to the microsatellite instability status of CRC (32). Interestingly, high levels of *F. nucleatum* improved the overall anti-tumor effects of PD-L1 blockade therapy with prolonged survival. Microbiome sequencing is another key topic identified in this analysis. Crucial alterations in carcinogenesis and treatment effects could be monitored by microbiota metagenomic profiling (33). 16S rRNA sequencing techniques have contributed to the identification of several key bacterial strains, including *F. nucleatum*, *E. coli*, *Streptococcus intermedius*, *Gemella haemolysans*, and *B. fragilis* (34). Another 16S rRNA amplicon sequencing study from Korea observed three phyla were less found in tumor tissues, along with intensely enriched metabolic pathways of the bile acid section or bacterial motility proteins related to CRC (35). In addition, *F. nucleatum* and *B. fragilis* were also found more abundant in recurrent individuals compared to nonrecurrent ones (35). Besides, based on the keywords heatmap (**Figure 3**), *F. nucleatum*, a gram-negative, anaerobic opportunistic bacterium, is most likely to be associated with CRC. This kind of bacterium is common to the oral cavity and is associated with periodontal disease. Interestingly, one of the topics identified by LDA algorithms is “patient oral health control,” covering studies of the association between oral health and CRC risk. Accumulating evidence has also concluded that mucosa-associated *Escherichia coli* (*E. coli*) is involved with the tumorigenesis and progression of CRC, particularly some strains of *E. coli*, including enteropathogenic *E. coli* (EPEC) and cyclomodulin-positive *E. coli* (13). Based on the topic terms, both *E. coli* and *Fusobacterium* are the main gut microbiota members associated with CRC.

Regarding microbiome compositions, interactions, and treatment, research is more likely to focus on human and animal models, treatment effects, inflammation bowel disease, immune responses to cancer immunotherapy, microbiome interactions in the host, microbiome dysbiosis, and infections. In fact, increasing experimental models have been developed to support rising research on microbiota in cancer studies, including a germ-free humanized microbiota sample transfer model and an antibiotic regiment-based animal model (**Table 2**). Effective animal models serve as an essential component of preclinical studies and critical evidence for mechanistic insights (36). Specific functional proteins such as FadA and Fap2 have been identified through successful experimental models (37–39). Interestingly, systemic, and mucosal immune status remains largely stable in the antibiotic treatment-based animal model, thereby making it a potential approach for immunotherapy evaluation. Soon, humanized microbiota animal models will be crucial to immunotherapy research (40–43) (**Table 2**). By far, studies are only beginning to scratch the surface of the nature of the association between the microbiome and CRC. It is reasonable to presume that microbiota-related treatment, immune response, and animal models will be some of the key developments in the future.

**Table 2:** Humanized microbiota-associated animal models.

Animal models	Stability	Features
Human microbiota samples transferring to germ-free animal model	Short term	1. Germ-free condition with specialized equipment 2. Expensive maintenance 3. Experimental genotyping 4. Developmental defects 5. Targeting specified microbes
Antibiotics treatment-based animal model	Potential long-term	1. Cost effective 2. Feasible to alternative types of antibiotics 3. Applicable to multiple genotypes 4. Bacterial or other microorganism bias to antibiotics treatment 5. Host health concerns in response to antibiotics treatment

Regarding microbiome molecular features and mechanisms, research topics are focused on regulation of pathway signals, expression of cytokines, colitis in mice, intestinal barrier, and gene and miRNA expression. Several pathway signals relating to tumorigenesis have been involved in the gut microbiota, including the toll-like receptors (TLRs), KRAS, NF-kappa B, SARS-CoV-2, G protein-coupled receptors, and Wnt pathways (44–49). Chronic inflammation was taken as a major cause of tumorigenesis and progression. Therefore, the colitis model has been an opportunity to reveal the contribution of microbiota to colitis-associated CRC (50).

Regarding the microbiome and metabolism, topics include activities of oxidative stress, metabolism, bile acids and butyrate, cell growth inhibition and potential effects of compounds, probiotics and prebiotics, dietary intake, and consumption. From a metabolic point of view, the microbiome and metabolism demonstrated a dramatic research market with huge potential. Bile acid–gut microbiome interaction constitutes one of the most intensively investigated topics, with increasing publications over the years (51–54). The metabolism of bile acids and their symbiosis was commonly associated with a low-fiber diet. Moreover, researchers indicated bidirectional regulatory effects of bile acids on CRC along with its progression (55). Butyrate, one of the main short-chain fatty acids, serves as an effector for anti-inflammation and anti-tumor (56). Increasing levels of omega-3 polyunsaturated fatty acids may promote the bacteria that produce butyrate, lowering the risk of CRC (57). Although the overall trend in the gut microbiome has been positive, some specific topics indicated that research processes were lagging, including dietary intake and consumption, pathogen strains, and activities of oxidative stress, all of which belong to this cluster.

The most influential reference was identified as “Intestinal inflammation targets cancer-inducing activity of the microbiota” by Arthur et al., published in SCIENCE 2012. In this study, Arthur et al. reported that *Escherichia coli NC101* (E. coli) was significantly enriched in inflammatory bowel disease and CRC mucosa. Intestinal microbiota was identified as a key target of intestinal inflammation, further affecting the disease course of CRC (58). This study for the first time answered the critical question of whether the gut microbiota was actively involved in the progress of carcinogenesis.

Among all the leading institutions, INRA showed the longest period in SCB. As one of the top research institutions in France, INRA has made a considerable contribution to the field of gut microbiota. To achieve deep knowledge of health-related challenges affected by the gut microbiota, INRA also launched a nationwide collaborative project called Le French Gut on 15 September 2022, continuing to support the “Million Microbiome of Humans Project,” an international project to build up a world-class human microbiota database for public use.

Of all the 50 topics, some showed remarkable progress during the last few years, for example, the role of colitis in a mouse model associated with gut microbiota and CRC. A few noticeable achievements had been made by several studies (59, 60). Zhu et al. reported that by precisely editing the microbiota composition in mouse models, the risk of tumor development in colitis-associated CRC could be reduced (59). Particularly, Enterobacteriaceae was identified as the key target in this intestinal inflammation course. By using a similar colitis cancer mouse model, Ji et al. highlighted the modulatory role of jujube polysaccharides in ameliorating colitis-related cancer and microbiota dysbiosis. Firmicutes and Bacteroidetes were also significantly reduced in this model (60).

Colitis-associated colorectal cancer (CAC) is a critical complication of inflammatory bowel disease, accounting for around 15% of mortality. However, the molecular mechanisms underlying the carcinogenesis of CAC remain largely unclear and are deemed different from other types of CRC (61). In fact, previous clinical and experimental clues have indicated that inflammation serves as a key to initiating CAC, while it may not be a decisive trigger for common CRC. Thus, investigation into the role of the gut microbiome in CRC and CAC is highly valuable. Research progress relating to gut microbiota and CAC is mostly represented by two types of studies. Type I is the mechanistic interaction between members of the microbial community and the intestinal tract. Microbial community members produce and release genotoxins in the gut to exasperate inflammation or induce carcinogenesis. Type II is the well-established animal model used for CAC. Of note, there is a high-value unanswered question: could an inflammatory bowel disease-driven microbe be able to automatically promote CAC and, in a broader sense, CRC? In fact, there are several types of microorganisms that are both linked to the disease courses of inflammatory bowel disease and CRC, including *Fusobacterium* species and *Streptococcus bovis*. Other types of microorganisms, such as the enterotoxic strain of *B. fragilis*, are only associated with CRC. Up until now, a disputate remains as to whether microbes are vital to the carcinogenesis of CAC and other types of CRC or just innocent bystanders. Animal models of CAC may be the key to that question.

## Conclusion

This bibliometric analysis explores the historical research trends in gut microbiome and CRC and identifies specific topics with increasing interests. The developmental trajectory, along with the noticeable research topics characterized by this analysis, will contribute to the future direction of research in CRC and clinical translation.

## Data availability statement

The original contributions presented in the study are included in the article/supplementary material. Further inquiries can be directed to the corresponding author.

## Author contributions

CY, ZZ, BL, DY, YH, and PW carried out literature and data analysis.CY, ZZ, BL, DY, YH, and YL drafted the manuscript. CY, ZZ, BL, DY, YH, PW, and YL participated in study design and data collection. All authors listed have made a substantial, direct, and intellectual contribution to the work and approved it for publication.
